# Screening Workers for Occupational Exposure to Respirable Crystalline Silica: Development and Usability of an Electronic Data Capture Tool

**DOI:** 10.2196/64111

**Published:** 2025-02-24

**Authors:** Fiona Hore-Lacy, Christina Dimitriadis, Ryan F Hoy, Javier Jimenez-Martin, Malcolm R Sim, Jane Fisher, Deborah C Glass, Karen Walker-Bone

**Affiliations:** 1 School of Public Health and Preventive Medicine, Faculty of Medicine, Nursing and Health Sciences, Monash University, Melbourne, Australia; 2 Department of Respiratory Medicine, Alfred Health, Melbourne, Australia

**Keywords:** silicosis, occupational history, electronic data capture tool (EDCT), REDCap, occupational respiratory screening, occupational hazard, exposure, silica, fibrotic lung disease, lung disease, respirable crystalline silica, mining, construction, workers, occupational lung disease, occupational, Australia, screening

## Abstract

**Background:**

Cases of the occupational lung disease silicosis have been identified in workers processing artificial stone in the stone benchtop industry (SBI). In the Australian state of Victoria, the Regulator commissioned a screening program for all workers in this industry.

**Objective:**

To facilitate systematic data collection, including high-quality exposure assessment, an electronic data capture tool (EDCT) was developed.

**Methods:**

A multidisciplinary team developed an EDCT using Research Electronic Data Capture (REDCap; Vanderbilt University). The needs of the EDCT were (1) data entry by multiple clinicians and the workers attending for screening and (2) systematic collection of data for clinical and research purposes. The comprehensibility and utility of the tool were investigated with a sample of workers, and the EDCT was subsequently refined.

**Results:**

The EDCT was used in clinical practice, with capacity for data extraction for research. Testing of comprehension and utility was undertaken with 15 workers, and the refined version of the Occupational Silica Exposure Assessment Tool (OSEAT) was subsequently developed.

**Conclusions:**

The refined OSEAT has been determined to be comprehensible to workers and capable of collecting exposure data suitable for assessment of risk of silicosis. It was developed for workers in the SBI in Australia and is adaptable, including translation into other languages. It can also be modified for SBI workers in other countries and for use by workers from other industries (mining, construction) at risk of silica exposure, including in lower-income settings.

## Introduction

Silicosis is an incurable, potentially life-threatening, form of fibrotic lung disease caused by inhalation of respirable crystalline silica (RCS) [1]. The disease has been recognized globally for over 100 years, and lung disease screening is recommended for high-risk industries, including mining and construction [2]. Cases of silicosis were identified in 2010 among workers in the stone benchtop (countertop) industry (SBI) working with artificial stone (AS; Textbox 1) [3-12]. Subsequently, a number of cases of artificial stone-associated silicosis were diagnosed in Australia [3,13]. AS has a very high crystalline silica content, often over 90% [14]. Processing AS by drilling, polishing, cutting, or grinding generates fine particles of dust containing RCS, which can cause silicosis when inhaled [15].

Textbox 1. **Timeline of artificial stone (AS) and silicosis in Australia**

Early 2000s: AS introduced to Australia

2010: First case of silicosis associated with AS reported in Italy

2015: First case of silicosis associated with AS reported in Australia (conference abstract)

2017: First case of silicosis associated with AS reported in Australia

2019: Screening program of stone benchtop industry workers begins in Victoria, Australia (paper-based data collection)

2021: Screening program first incorporates electronic data capture tool (EDCT)

2021-2023: Refinement of EDCT informed by data cleaning and evaluation study with workers

An investigation of the effects of RCS exposure in the SBI was commissioned by the Victorian regulator, WorkSafe Victoria [16], and developed into a screening program by Monash University. It included (1) exposure assessment from a detailed occupational history; (2) collection of respiratory symptoms; (3) recording of investigations including spirometry, chest x-ray, and high-resolution CT of the chest; (4) screening for comorbidities associated with silica exposure, including autoimmune diseases and tuberculosis [4,17]; and (5) a mental health instrument.

The initial paper-based questionnaire was developed by a multidisciplinary team, including respiratory physicians, an occupational hygienist, and occupational physicians [16]. Simplicity was prioritized, as many industry workers were born outside Australia and spoke English as an additional language [18].

Up to 6 jobs in the SBI could be recorded in the occupational history. The proportion of time spent on specified *tasks* in each job and the proportion of time spent on dry cutting of stone and working near someone doing dry cutting were recorded. *Exposure control measures* (ventilation and respirator use) were identified for each job. Other information collected included the country, start and (if relevant) finish date; days per week worked; number of people in the organization; and type of stone predominantly worked with (AS or natural stone). Other silica-associated occupations (eg, mining, quarrying) and any non-occupational activities that involved dust exposure (including hobbies and home repairs, eg, tiling, plastering) were also recorded.

Data were collected from multiple users, including respiratory physicians, multidisciplinary team, workers, and administrative staff, and capture all the elements listed earlier. The data were cleaned and entered into an electronic data capture tool (EDCT) held on the secure REDCap platform [19,20]. The data were used for both clinical and research purposes. In 2021, screening was centralized and carried out at a single site, which led to a need for direct data entry to the EDCT by the worker.

Exposure calculations from the occupational data have been used to identify roles within the SBI with greater RCS exposure, such as factory machinists and installers, and that exposure intensity and cumulative exposure were associated with dyspnea and radiological abnormalities consistent with silicosis [21]. The screening program data has also been used to describe the numbers of cases of silicosis diagnosed to date [18], the rates and determinants of psychological distress, and the psychometric properties of the mental health instrument [22,23].

The aim of this study was to describe the development of the EDCT, present its refined content, and describe the results of an audit of its clinical utility undertaken with a sample of workers.

## Methods

### Overview

The original team reviewed the exposure questionnaire, which overall had been well understood, and identified items that required substantial data cleaning. The team redeveloped the questions, which included, for example, adding illustrations of dry and wet cutting examples and the types of respirator and ventilation options that were sourced from workplace health and safety organizations [24-26]. Further, additional optional responses were added from free text replies, for example, water jet cutting.

**Figure 1.**
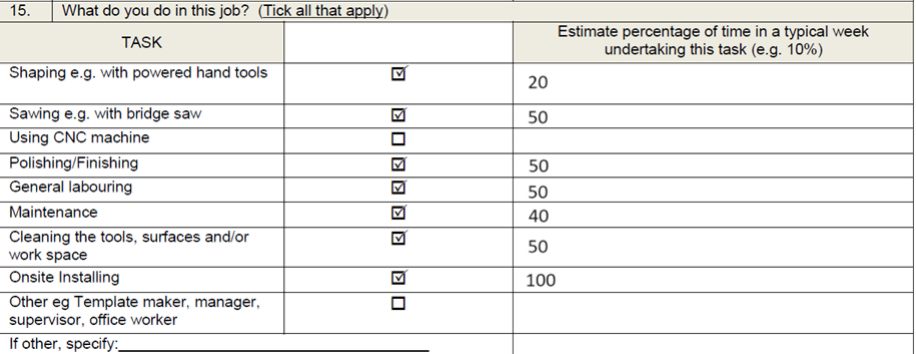
Original paper questionnaire asking workers to estimate proportions of work time spent doing specific tasks in their workplace. CNC: Computer Numerical Control____.

In the first draft questionnaire, participants were asked to apportion their tasks (Figure 1).

**Figure 2.**
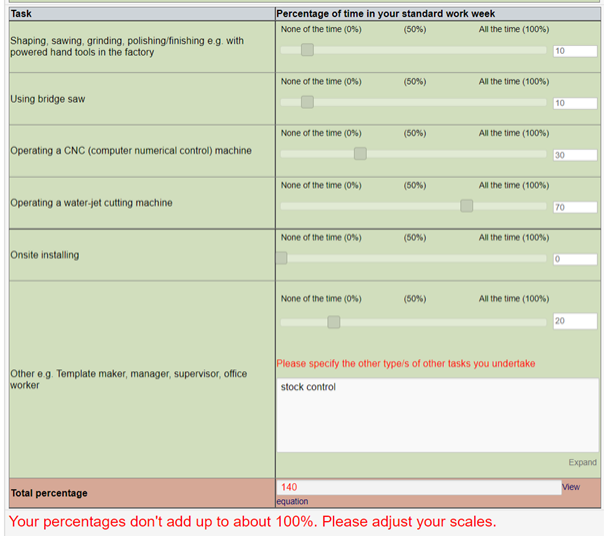
EDCT (electronic data capture tool) version of the task estimation section of the occupational history with a total that adds to a proportion of >100% of the time and the warning message provided to the worker.

The percentages seldom added to 100%, as shown in the example in Figure 1, so in the revised questionnaire, a sliding bar was provided that provided visual input of the proportions (Figure 2). A pop-up trigger was included if the task proportions were out of range, as shown in Figure 2.

Prior to attending for screening, workers were emailed a link to the EDCT containing the revised questions in order to complete their occupational history. The final page completed by workers included optional electronic consent for sharing data with Monash University.

Other data collected during screening included medical history, smoking status, respiratory symptoms, physical examination findings, diagnosis, return-to-work assessment, and results of all relevant investigations, including chest x-ray, high-resolution CT, pathology, and spirometry.

In 2023, an investigation of the comprehensibility, feasibility, and face validity of the occupational history section of the EDCT was completed with workers.

A total of 15 workers participated in the investigation, which was conducted between February and May 2023. Workers were interviewed about their responses on the EDCT, using a pre-developed proforma to prompt feedback. They were asked: “Were there any words you did not know?” “Did you understand what was meant by ‘dry work’” “Are there any other tasks in your workplace that expose you to dust?” and “Did the list of ventilation options include what you use in your workplace?” For all questions, respondents could reply yes or no and provide additional comments. If a worker reported no dry cutting in their current or most recent job but had exposure to dry cutting from previous jobs, they were asked to respond to questions referring to their former job.

#### Ethical Considerations

Workers were eligible to participate if they had completed their occupational history using the EDCT, did not require an interpreter for their appointment, and had provided electronic consent to share data with Monash University. All participants were provided with a written information sheet and asked to provide verbal consent for participation. Responses were deidentified, and workers did not receive compensation for their participation. Approval was granted by the Alfred Hospital Research Ethics Committee as a substudy of project 292/21.

## Results

The demographics of the workers are presented in Table 1. Most participants were male, and they covered a range of ages and years of employment in the SBI. They included machinists, installers, and office workers.

**Table 1.** Demographics of participants.

Characteristics		Values
Males, n (%)		14 (93)
Age (years), mean (SD)		38.1 (10.9)
Years in stone benchtop industry, mean (SD)		9.6 (7.85)
Born in Australia, n (%)		5 (33)
Language other than English spoken at home, n (%)		6 (40)
Most recent SBI job held, n		
	Director	3
	Installer	4
	Stonemason	5
	Foreman	2
	Other	1

All participants were asked to report about their *comprehension* of an introductory statement, and one worker commented that it took a while to read and understand, and did not feel like he understood it fully. All workers reported that they comprehended what was meant by “dry work.” When asked about *tasks* that exposed them to dust that were not already listed on the EDCT, 2 workers identified new relevant tasks: emptying bins containing benchtop fragments and cleaning of the final benchtop product, onto which the dust-containing water used in wet cutting had dried. In total, 4 workers reported that their *ventilation* option was not included in the list on the EDCT, nor was it pictured. However, after discussion, the alternative options they were describing were “air conditioning,” “garage door,” “no ventilation,” and “ventilation in the wall,” all of which were listed.

For *respirators*, 14 workers identified the type they used from the descriptions and pictures in the EDCT, and the only worker that did not see their device described use of one similar to that depicted. Of 8 workers who were asked if they could easily remember and estimate the percentage of time they spent wearing a respirator, 7 responded in the affirmative. One worker had worn his respirator for 6 hours out of an 8-hour shift (approximately 75%), but had estimated that he wore it for 35% of the day. One worker commented that he wore his respirator all day, regardless of the task, whilst another pointed out that each job was different, with some jobs being “perfect” (ie, not requiring any adjustments), whereas others required adjustments onsite, adding to the difficulty of responding to this question accurately.

If a worker reported dry cutting in their current job, they were asked whether or not practices around dry cutting had changed (this was because dry cutting without suitable protection had been officially banned recently in Australia). Of the 11 workers who completed this question, 10 understood the question and were able to complete it accurately, but one worker expressed some confusion around the wording of the question. Workers also described measures other than ventilation and respirators that their employer had introduced including changing clothes at work and using water systems for dust suppression when loading stone.

**Figure 3.**
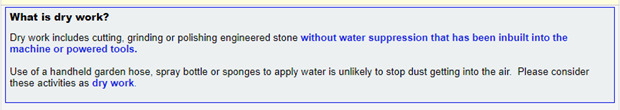
Refined EDCT for clarification of the definition of dry work.

Subsequently, modifications were made to improve accessibility for the workers: simplification of the language used in descriptive statements and instructions, modification of the color scheme to improve readability, and addition of commonly reported responses (eg, “home maintenance,” “tiling,” and “plastering”) as a prompt on the non-occupational (eg, hobby) dust exposure history section. One of the simplified statements was an introductory statement that defined “dry work” (Figure 3). An image accompanied the text with examples of dry and wet cutting [26]:

The current version of the Occupational Silica Exposure Assessment Tool from the EDCT is presented in Multimedia Appendix 1.

## Discussion

### Principal Findings

Since 2019, over 1000 SBI workers have undergone screening for silicosis in Victoria, Australia, through a protocolized screening program. In this study, we described the development of the screening questionnaire into an EDCT, the Occupational Silica Exposure Assessment Tool, and have described how it was refined as a result of our experiences and after assessment of its acceptability and comprehension among this worker population. The results of the investigation suggested that the usability and comprehension of the refined EDCT are acceptable among English-speaking workers.

The benefits of EDCTs for improving patient care [27]; improving accuracy of data collection compared to paper methods [28]; and facilitating data collection from multiple users, including patients and health care providers [29], have been established in many settings. Moreover, EDCTs are able to capture and retain large volumes of data, maximizing cost- and time-efficiency in clinical and research settings [29,30]. As demonstrated, the development of this EDCT has already provided all of these benefits and enabled us to create a streamlined and efficient screening program for workers in the artificial stone benchtop industry.

Benchtops made from AS are a popular kitchen product globally, and there are concerns that cases of silicosis among workers who produce them are underreported in the literature [31,32]. Globally, silica deaths were estimated to be more than 12.9 thousand in 2019 [33], and the highest rates were recorded in low- and middle-income countries [33,34]. Silicosis has been seen in a range of industries, including construction, jewelry production, quarrying, tunneling, dental material manufacturing, denim jean production, and ceramic and pottery manufacturing [35]. There is therefore an urgent need for occupational screening for large numbers of workers exposed to RCS, for which reliable instruments are needed. The Occupational Silica Exposure Assessment Tool can be deployed in settings in which workers are exposed to RCS, whether for workers in the SBI or modified for other occupational settings.

In addition to assessing SBI workers at risk of silicosis, the data collected from the Occupational Silica Exposure Assessment Tool (OSEAT) can be used to estimate an individual’s level of RCS exposure. In previous work, occupational history data collected using the OSEAT were used to group SBI workers by extent of silica exposure, using a combination of the proportion of time working with AS and the proportion of time spent dry cutting [21]. Both cumulative exposure and exposure intensity were found to be associated with symptoms of dyspnea and chest x-ray abnormalities [21]. This illustrates the ready utility of having added the e-consent function to the EDCT within REDCap, facilitating extracting data from the OSEAT to use for research purposes.

One consideration when introducing a REDCap-based EDCT in a clinical setting is its reliance on workers having adequate internet connection. This has been a limiting factor for the utilization of similar instruments, in which open-source software that was not reliant on internet connection was preferred [28,36]. In some resource-poor settings, REDCap was the preferred platform [37,38], and REDCap has now developed a mobile app that can be used offline that may overcome internet connection limitations [20].

A limitation of this study was the small number of workers included, none of whom required interpreters. Its comprehensibility is therefore unknown among those who require interpreters. Furthermore, these workers were recruited consecutively and recently from the clinic and consequently are not necessarily representative of the wider workforce. Another limitation was that the original instrument was not co-designed with consumers (workers in the SBI), something that we addressed in this study. We were unable to investigate floor or ceiling effects within the scope of this work, which would be a necessary step if the tool is to be used in translated versions.

A strength of the OSEAT is that it has been used and improved for close to 3 years with demonstrably good comprehension by SBI workers.

Future development of the OSEAT will include its translation into other languages using the REDCap multilanguage module. The commonest languages other than English among the workers attending screening at our center have been Vietnamese, Persian, Chinese, and Arabic, and are therefore a priority for translation and inclusion into the module.

## Conclusions

This study has presented the development, comprehension, utility, and refinement of the OSEAT, a purpose-built EDCT for use among SBI workers undergoing assessment for silicosis that included input from workers and has the capacity for modification and use within other silica-exposed occupational settings.

## Supplementary material

10.2196/64111 Multimedia Appendix 1 Occupational history data collection template: stone benchtop industry, and other silica and nonsilica exposed jobs.

## Abbreviations

AS: artificial stone
CNC: Computer Numerical Control
CT: computed tomography
EDCT: electronic data capture tool
OSEAT: Occupational Silica Exposure Assessment Tool
RCS: respirable crystalline silica
REDCap: Research Electronic Data Capture
SBI: stone benchtop industry
