# Supplementary material for: Screening Workers for Occupational Exposure to Respirable Crystalline Silica: Development and Usability of an Electronic Data Capture Tool
Source: JMIR Hum Factors. 2025 Feb 24;12:e64111. doi: 10.2196/64111 (PMC11875104; doi:10.2196/64111)
Supplement: Multimedia Appendix 1 [file humanfactors-v12-e64111-s001.pdf]

Appendix A: Occupational history data collection template: stone benchtop industry and other silica and non-silica exposed jobs.

Hore-Lacy et al. Screening of workers for exposure to respirable crystalline silica: development of the Occupational Silica Exposure Assessment Tool, an electronic data capture tool, and establishment of its clinical utility.

Stone benchtop industry summary

Year started work in the stone benchtop industry	2014
Are you still working in the stone benchtop industry?	(●) yes ( ) no reset
If No, what year did you finish work in the stone benchtop industry?	
If no, which of the following best describes you?	If other, please specify
How many different workplaces/employers have you had in the stone benchtop industry?	2
How many different jobs have you had in the stone benchtop industry?<br><br>• Please also include unpaid work in the benchtop industry, such as helping in family businesses or apprenticeships<br>• If you have changed jobs within the same business, please record this as separate jobs<br>• If you have left a business and returned to the same business, please record this as two separate jobs.	2
During your time in the stone benchtop industry have you worked mainly in the factory or been involved in the processes of onsite installations?	( ) Onsite installations<br>(●) Factory-based (including office work)<br>reset

We want to know about your jobs in the **Stone Benchtop Industry:**

- **List a maximum of six jobs**
- **If more than six, enter the six longest jobs**
- **Start with your current or most recent job**
- If you have **changed jobs within the same business,** please record this as **two separate jobs.** (For example, if you started as a machinist and became a supervisor)
- If you are unsure of the date, **please estimate.**

In the next section, we ask about:

- Other silica-associated jobs (Cathedral or monumental masonry, tiling)
- All other jobs, regardless of industry
- Hobbies with dust exposure

Job number	What is the business name?	What is/was your job title at this business?	What date did you start at this business?	Is this job current?	What date did you finish at this business?
Current / most recent job	Sample company 1	Stonemason	01-01-2020<br>D-M-Y	(●) Yes<br>( ) No<br>reset	

Previous jobs

2	Sample company 2	Installer	01-01-2014 D-M-Y		31-12-2019 D-M-Y

What is dry work?

Dry work includes cutting, grinding or polishing engineered stone **without water suppression that has been inbuilt into the machine or powered tools.**

Use of a handheld garden hose, spray bottle or sponges to apply water is unlikely to stop dust getting into the air. Please consider these activities as **dry work**.

An image that contains examples of dry and wet cutting is used here.

When was the last time you worked in an environment where there was dry processing?<br>This includes when you have personally done dry or have been working near a person undertaking dry processing.<br>If you do not know the exact day please enter '01'. If you do not know the month please estimate.	01-01-2019 D-M-Y<br>☐ never

FURTHER JOB HISTORY DETAILS

Job number 1: Current job

Business name: Sample company 1

Job title: Stonemason

Start date: 01-01-2020 to current

Which country is the business in?	Australia
If in Australia which state is the business in?	VIC
How many people work/ed in the business?	◉ < 10<br>○ 10 - 50<br>○ 50 - 250<br>○ Over 250<br>reset
Please specify the number of days a week you work/ed?	5

Thinking about your overall time at the Sample company 1, please select the option that best describes the type of stone used.	(●) Only uses artificial stone<br>( ) Uses between 50%-99% artificial stone<br>( ) Uses less than 50% artificial stone<br>( ) Only uses natural stone<br>reset
If you use/used stone other than artificial stone at this business please select the main type you used? Please select one.	

Task information

We would like to ask about the type of tasks you currently do in your standard work week at Sample company 1 in your job as a Stonemason.

Below is a list of tasks that are common in the stone benchtop industry. Please tell us the percentage of time you do these tasks in your standard work week using the sliders.

If you do not do the task, please move the slider to "none of the time". If your standard tasks are not listed please select "other" and tell us the types of tasks in the text box provided.

Once you have selected your standard tasks and allocated the percentage of time for each task, the total should equal 100% .

Task	Percentage of time in your standard work week
Shaping, sawing, grinding, polishing/finishing e.g. with powered hand tools in the factory	None of the time (0%) (50%) All the time (100%) 10 reset
Using bridge saw	None of the time (0%) (50%) All the time (100%) 5 reset
Operating a CNC (computer numerical control) machine	None of the time (0%) (50%) All the time (100%) 37 reset
Operating a water-jet cutting machine	None of the time (0%) (50%) All the time (100%) 25 reset
Onsite installing	None of the time (0%) (50%) All the time (100%) 0 reset

Other e.g. Template maker, manager, supervisor, office worker	None of the time (0%) (50%) All the time (100%) 21 reset Please specify the other type/s of other tasks you undertake: Template maker Expand
**Total percentage**	98

Your percentages add up to about 100%

Ventilation use

In your job as a Stonemason at Sample company 1 do you currently use any type/s of ventilation?

Types of ventilation include:

- extraction on the bench
- extraction on the tools
- extraction booth
- fan (pedestal, in the wall, ceiling etc)
- open door/window
- other type/s of ventilation

If you use ventilation that is not listed please select "yes" and specify the "other" type of ventilation in the text box provided.

Please see images below for some examples.

(•) Yes ( ) No

reset

An image that contains examples of ventilation options is used here.

If yes, please select the types of ventilation you currently use in your job as Stonemason, at Sample company 1.

Select all that apply

- [x] extraction on the bench
- [x] extraction on the tools
- [ ] extraction booth
- [x] fan (pedestal, in the wall, ceiling etc)
- [x] open door/window
- [ ] other type/s of ventilation

Respirator Use

In your job as Stonemason at Sample company 1 do you currently use any type/s of respirator/s (face mask)?

Types of respirators include:
- Paper/disposable half face respirator
- Reusable half face respirator
- Full face respirator
- Soft top hood/helmet with air supply
- Half face with air supply
- Full face with air supply

Refer to images below for examples.

(●) Yes ( ) No

reset

An image that contains examples of respirator options is used here.

If yes, please select the type/s of respirator/s you currently use in your job as Stonemason at Sample company 1. Select all that apply

- [ ] Paper/disposable half face respirator
- [ ] Reusable half face respirator
- [ ] Full face respirator
- [ ] Soft top hood/helmet with air supply
- [x] Half face with air supply
- [ ] Full face with air supply

What proportion of time do you currently wear a respirator in your job as a Stonemason, at Sample company 1?	None of the time (0%) (50%) All of the time (100%) 76 reset

Dry cutting/work practices

During your time as a Stonemason, has Sample company 1 reduced the amount of dry work or stopped dry work?	( ) Yes, my workplace has reduced or stopped dry work<br>( ) No, there have been no changes<br>(●) Dry work has never been performed during my employment period<br>reset

FURTHER JOB HISTORY DETAILS

Job number 2

Business name: Sample company 2

Job title: Installer

Start date: 01-01-2014

End date: 31-12-2019

Which country is the business in?	Australia
Which state is the business in?	VIC
How many people work/ed in the business?	(●) < 10<br>( ) 10 - 50<br>( ) 50 - 250<br>( ) Over 250<br>reset
Please specify the number of days a week you work/ed?	5
Thinking about your overall time at the Sample company 2, please select the option that best describes the type of stone used.	( ) Only uses artificial stone<br>(●) Uses between 50%-99% artificial stone<br>( ) Uses less than 50% artificial stone<br>( ) Only uses natural stone<br>reset
If you use/used stone other than artificial stone at this business please select the main type you used? Please select one.	( ) Marble<br>(●) Granite<br>( ) Travertine<br>( ) Sandstone<br>( ) Quartzite<br>( ) Dolomite<br>( ) Bluestone<br>( ) Other<br>reset

Task Information

We would like to ask about the type of tasks you did in your standard work week at **Sample company 2** during your time as a **Installer.**

Below is a list of tasks that are common in the stone benchtop industry. Please tell us the percentage of time you did these tasks in your standard work week using the sliders.

If you did not do the task, please move the slider to "none of the time". If your standard tasks are not listed please select "other" and tell us the types of tasks in the text box provided.

Once you have selected your standard tasks and allocated the percentage of time for each task, the total should equal **100%.**

Task	Percentage of time in your standard work week
Shaping, sawing, grinding, polishing/finishing e.g. with powered hand tools in the factory	None of the time (0%) (50%) All the time (100%) 0 reset
Using bridge saw	None of the time (0%) (50%) All the time (100%) 0 reset
Operating a CNC (computer numerical control) machine	None of the time (0%) (50%) All the time (100%) 0 reset
Operating a water-jet cutting machine	None of the time (0%) (50%) All the time (100%) 0 reset
Onsite installing	None of the time (0%) (50%) All the time (100%) 100 reset
Other e.g. Template maker, manager, supervisor, office worker	None of the time (0%) (50%) All the time (100%) 0 reset Please specify the other type/s of other tasks you undertake
**Total percentage**	100

Your percentages add up to about 100%

Ventilation use

In your job as Installer at Sample company 2 did you use any type/s of ventilation?

Types of ventilation include:

- extraction on the bench
- extraction on the tools
- extraction booth
- fan (pedestal, in the wall, ceiling etc)
- open door/window

- other type/s of ventilation

If you used ventilation that is not listed please select "other" and specify the type of ventilation in the text box provided.

(•) Yes ( ) No

reset

An image that contains examples of ventilation options is used here.

If yes, please select the types of ventilation you used in your job as Installer at Sample company 2. Select all that apply

- [ ] extraction on the bench
- [x] extraction on the tools
- [ ] extraction booth
- [ ] fan (pedestal, in the wall, ceiling etc)
- [ ] open door/window
- [ ] other type/s of ventilation

Respirator use

In your job as Installer at Sample company 2 did you use any type/s of respirator/s (face mask)?

Types of respirators include:
- Paper/disposable half face respirator
- Reusable half face respirator
- Full face respirator
- Soft top hood/helmet with air supply
- Half face with air supply
- Full face with air supply

Refer to images below for examples.

(•) Yes ( ) No

reset

An image that contains examples of respirator options is used here.

If yes, please select the type/s of respirator/s you used in your job **Installer**, at **Sample company 2**. Select all that apply

- [ ] Paper/disposable half face respirator
- [ ] Reusable half face respirator
- [ ] Full face respirator
- [x] Soft top hood/helmet with air supply
- [ ] Half face with air supply
- [ ] Full face with air supply

What proportion of time did you wear a respirator in your job as **Installer**, at **Sample company 2**?	None of the time (0%) — (50%) — All of the time (100%) 32 reset

Dry cutting/work practices

During your time as a **Installer,** has **Sample company 2** reduced the amount of dry work or stopped dry work?	(●) Yes my workplace has reduced or stopped dry work ( ) No, there have been no changes ( ) Dry work has never been performed during my employment period reset

If yes, what date did **Sample company 2** reduce the amount of dry work or stop dry work? If you **do not know** the day enter **01** as the day, and if you are unsure of the month and year please estimate.	01-01-2019 D-M-Y

AFTER changes in dry work on **01-01-2019** at **Sample company 2**

Estimate the proportion of time you did dry work?	None of the time (0%) — (50%) — All of the time (100%) 11 reset
Estimate the proportion of time you were **near someone else** doing dry work?	None of the time (0%) — (50%) — All the time (100%) 0 reset

BEFORE changes in dry work on **01-01-2019** at **Sample company 2**

Estimate the proportion of time you did dry work?	None of the time (0%) — (50%) — All of the time (100%) 50 reset
Estimate the proportion of time you were **near someone else** doing dry work?	None of the time (0%) — (50%) — All of the time (100%) 50 reset

Other occupational history and hobbies

On this page, we would like to know about any additional jobs and hobbies you may have. These include:

1. Other silica-associated jobs
2. All other jobs, regardless of industry
3. Hobbies with dust exposure

1. Other silica-associated jobs

We would like to know more about **other jobs** you have had with **possible silica dust exposure** that you have not listed previously. This includes working with stone, clay, brick, concrete or porcelain in industries such as:

- Cathedral or monumental masonry
- Construction
- Tiling
- Mining
- Quarrying
- Tunnelling
- Concreting
- Masonry
- Pottery
- Foundries

Please only include jobs that you have held for more than 6 months. You can enter up to six other jobs. **Please do not list any jobs that you have listed in the job history section previously.**

Have you had any other jobs where you were possibly exposed to silica dust? ○ Yes ◉ No

reset

2. All other jobs

We would like to know **ALL other jobs** you have had.

This includes **jobs without silica exposure, regardless of industry**. Please do not list any jobs that you have listed previously. Please only include jobs that you have held for more than 6 months

Have you had any other jobs that are not listed in your job history? ◉ Yes ○ No

reset

If yes, please list all other jobs that you have not previously described.

Carpenter apprenticeship

Expand

3. Hobbies

We would like to know about any **hobbies that expose you to dust.**

This includes hobbies with silica or other dust exposure, regardless of industry. Examples include:

- Home maintenance (eg plastering, tiling, concreting)
- Pottery or ceramic work
- Stone work
- Surfboards
- Fibreglass.

Please do not list any jobs that you have listed previously. Hobbies can be current or ones that you have stopped.

Do you have any hobbies which involve exposure to dust?
* must provide value

(●) Yes ( ) No

reset

If Yes, please specify
* must provide value

Home and garden maintenance - tiling and concrete paving

Expand
